# Joint Demosaicing and Denoising Based on a Variational Deep Image Prior Neural Network

**DOI:** 10.3390/s20102970

**Published:** 2020-05-24

**Authors:** Yunjin Park, Sukho Lee, Byeongseon Jeong, Jungho Yoon

**Affiliations:** 1 Department of Mathematics, Ewha W. University, Seoul 03760, Korea; yunjjin92@gmail.com; 2 Division of Computer Engineering, Dongseo University, Busan 47011, Korea; petrasuk@gmail.com; 3 Institute of Mathematical Sciences, Ewha W. University, Seoul 03760, Korea; bjeong_ewha@ewha.ac.kr

**Keywords:** color filter array, deep image prior, demosaicing, deep learning

## Abstract

A joint demosaicing and denoising task refers to the task of simultaneously reconstructing and denoising a color image from a patterned image obtained by a monochrome image sensor with a color filter array. Recently, inspired by the success of deep learning in many image processing tasks, there has been research to apply convolutional neural networks (CNNs) to the task of joint demosaicing and denoising. However, such CNNs need many training data to be trained, and work well only for patterned images which have the same amount of noise they have been trained on. In this paper, we propose a variational deep image prior network for joint demosaicing and denoising which can be trained on a single patterned image and works for patterned images with different levels of noise. We also propose a new RGB color filter array (CFA) which works better with the proposed network than the conventional Bayer CFA. Mathematical justifications of why the variational deep image prior network suits the task of joint demosaicing and denoising are also given, and experimental results verify the performance of the proposed method.

## 1. Introduction

Nowadays, many digital imaging systems use a single monochrome sensor with color filter array (CFA) to capture a color image. Without color filters, the monochrome camera sensor would give only brightness or luminance information and could not recover the colors of the light that fall on each pixel. To obtain color information, every pixel is covered with a color filter that only lets through a certain color of light: red, green or blue. These sampled red, green and blue channels are then interpolated to fill in the missing information at the pixels for which certain colors could not be sampled. This procedure is called the demosaicing procedure, for which many methods have been proposed [1,2,3,4,5,6,7,8,9].

Current image sensors come in CCD (charge-coupled device) or CMOS (complementary metal–oxide–semiconductor) types, which are both sensitive to thermal noise. Therefore, CFA pattern images taken in low illumination suffer from noise of Poisson distribution. The noise in the CFA pattern image has a large effect on the reconstruction of the color image, as the noise in the noisy pixels spreads out to neighboring regions by the demosaicing process. The denoising is also a challenging task since at least two-thirds of the data are missing. Complex aliasing problems can occur in the demosaicing process if a poor denoising is applied beforehand. As most digital camera pipelines are sequential, quite often the demosaicing and denoising are also performed in an independent and sequential way. This leads to an irreversible error accumulation, since both the demosaicing and the denoising are ill-posed problems and the error occurring in one of the procedures cannot be undone in the other procedure. It has been shown that simultaneous coping with the errors in both the denoising and the demosaicing has advantages, and some joint demosaicing and denoising methods based on optimization techniques have been developed [10,11,12]. Recently, inspired by the success of the convolutional neural network (CNN) in many image processing tasks, methods which use the CNN in joint demosaicing and denoising have been proposed [13,14,15]. The work of [13] is a first attempt to apply a learning approach for demosaicing, and the work in [14] is a first attempt to use a convolutional network for joint demosaicing and denoising, but works only on a single noise level. The work in [15] exposes a runtime parameter and trains the network so that it adapts to a wider range of noise levels, but still can only work with a relatively low level of noise. The work of [16] proposes a residue learning neural network structure for the joint demosaicing and denoising problem based on the analysis of the problem using sparsity models, and that of [17] presents a method to learn demosaicing directly from mosaiced images without requiring ground truth RGB data, and showed that a specific burst improves the fine-tuning of the network. Furthermore, the work in [18] proposes a demosaicing network which can be described as an iterative process, and proposes a principled way to design a denoising network architecture. Such CNN-based methods need a lot of data to be trained, and normally, work poorly with varying noise.

In this paper, we propose a deep image prior based method which needs only the noisy image as the training data for the demosaicing. The proposed method uses as the input a sum of a constant and varying noise. We give mathematical justifications as to why the added varying input noise results in the denoising of the demosaiced image. Furthermore, we propose a color filter array which suits the proposed demosaicing method and show experimentally that the proposed method yields good joint demosaicing and denoising results.

## 2. Related Works

The following works are related to the proposed method. The proposed method can be seen as a variation of the following works fitted to the joint demosaicing and denoising problem.

### 2.1. Deep Image Prior

Recently, in [19], a deep image prior (DIP) has been proposed for image restoration. The DIP is a type of convolutional neural network which resembles an auto encoder, but which is trained with a single image $x_{0}$; i.e., only with the image to be restored. The original DIP converts a 3D tensor z into a restored image $f_{\theta }\left(z\right)$, where $f_{\theta }\left(\cdot \right)$ denotes the deep image prior network with parameters θ. The tensor z is filled with random noise from a uniform distribution. The DIP can be trained to inpaint an image with a loss function as follows:

(1) $$L=D\left(m⊙f_{\theta }\left(z\right),m⊙x_{0}\right),$$

where $m\in {\left\{0,1\right\}}^{H\times W}$ is a binary mask with values of zero corresponding to the missing pixels to be inpainted and values of one corresponding to the existing pixels which have to be kept, and ⊙ is an element-wise multiplication operator. Here, *D* is a distance measure, which is normally set as the square of the $L_{2}$ difference operator; i.e., $D\left(a,b\right)={∥a-b∥}_{2}^{2}$. The minimization of *L* in Equation (1) with respect to the parameters θ of the DIP network has been shown to be capable of inpainting an image; i.e., the minimization results in an inpainted image $f_{\theta }\left(z\right)$. Inpainting and demosaicing are similar in that they try to fill in missing pixels. The difference is that in inpainting the existing pixels have full channel information, i.e., all R, G and B values are available, whereas in the demosaicing the existing pixels have only one of the R, G and B values.

### 2.2. Variational Auto Encoder

The variational auto-encoder [20] is a stochastic spin of the auto-encoder which consists of an encoder $q_{\theta }\left(z|x\right)$ and a decoder $p_{\phi }\left(x|z\right)$, where both the encoder and decoder are neural networks with parameters θ and ϕ, respectively. Given an image x as the input, the encoder $q_{\theta }\left(z|x\right)$ outputs parameters to a Gaussian probability distribution. After that, samples are drawn from this distribution to get a noise input z to the decoder $p_{\phi }\left(x|z\right)$. The space from which z is sampled is stochastic and of lower dimension than the space of x. By sampling different samples each time, the variational auto-encoder learns to generate different images. The proposed method also samples noise from a Gaussian distribution, but, unlike the variational auto-encoder, the noise is constituted of constant noise and varying noise and is an input into the network and not used as an intermediate input stage to the decoder.

## 3. Variational Deep Image Prior for Joint Demosaicing and Denoising

In this section, we propose a variational deep image prior (DIP) for joint demosaicing and denoising. We denote by $f_{\theta }$ the DIP network, and use the same network structure as the DIP for inpainting as defined in [19]; i.e., a U-Net type network which is downsampled five times and upsampled five times. The loss function for the variational DIP differs from that of the original DIP as follows:

(2) $$L=\left\{\begin{array}{cc} ∥m_{r}⊙\left(f_{\theta }\left(z_{c}\right)-y^{k}\right){∥}^{2}] & \mathrm{for}\;k<P\\ ∥m_{r}⊙\left(f_{\theta }\left(z_{c}+z_{v}\right)-y^{k}\right){∥}^{2}] & \mathrm{for}\;k\geq P. \end{array}\right.$$

Here, $z_{c}$ and $z_{v}$ denote the constant noise and the varying noise, respectively, both derived from a Gaussian distribution, and $m_{r}$ is the binary mask corresponding to the proposed random CFA; i.e., it consists of three channels. Each channel constitutes of 33% of random pixels with the value one and 66% of random pixels having the value zero. Unlike the inpainting problem, the positions of the pixels having the value one are different for each channel. The input to $f_{\theta }$ is a constant noise ($z_{c}$) until the (P−1)-th training step. Then, after the (P−1)-th training step, the input becomes the sum of a constant noise ($z_{c}$) and a varying noise ($z_{v}$), where the noise $z_{v}$ is newly generated and differs for each training step. The effect of adding this varying noise $z_{v}$ will be explained later. The target image $y^{k}$ also differs for the different iteration steps:

(3) $$y^{k}=\left\{\begin{array}{cc} x_{0} & \mathrm{for}\;k<P\\ \underset{y}{\mathrm{argmin}}[\left(1-\alpha -\beta \right)∥m_{r}⊙\left(y-y^{k-1}\right){∥}^{2}+\alpha {∥m_{r}⊙\left(y-x_{0}\right)∥}^{2}\\ +\beta ∥m_{r}⊙\left(y-f_{\theta }\left(z_{c}\right)\right){∥}^{2}] & \mathrm{for}\;k\geq P \end{array}\right.$$

**Figure 1:**
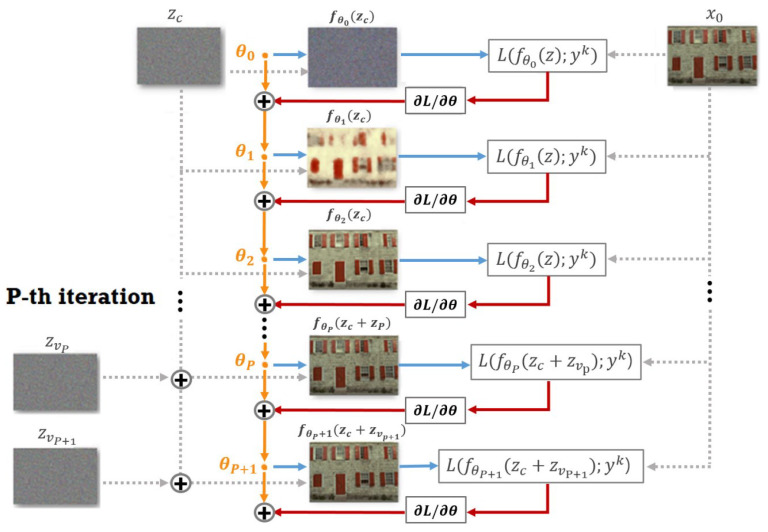
Diagram of the proposed method. The variational noise $z_{v_{P}}$, $z_{v_{P+1}},\ldots$ is added after the (P-1)-th iteration.

For the steps k<P, $y^{k}=x_{0}$, $x_{0}$ is a three channel image, wherein each channel contains 33% of either the R, G or B intensity values at the pixel positions where the R, G and B values are sensed by the random CFA, respectively, and 66% of zero values at the remaining positions. Furthermore, we assume that $x_{0}$ contains noise. Therefore, if $y^{k}=x_{0}$ for all steps *k*, then the reconstructed image $f_{\theta }$ will converge to a noisy demosaiced image. To avoid this, after the step k=P, the target image $y^{k}$ becomes a weighted average of the previous target image $y^{k-1}$, the given noisy image $x_{0}$ and $f_{\theta }\left(z_{c}\right)$. The weights between these images are controlled by α and β. In the experiments, we let α=0.003 and β=0.007 for all images. It should be noted that $f_{\theta }\left(z_{c}\right)$ differs from the current output $f_{\theta }\left(z_{c}+z_{v}\right)$. The image $f_{\theta }\left(z_{c}\right)$ is a denoised version of $y^{k-1}$, and the adding of it denoises the target image $y^{k}$. The adding of $x_{0}$ has the effect of adding back the noise, a trick which is widely used in denoising algorithms to restore the fine details back to the target image. The adding of the previous target image $y^{k-1}$ keeps the record of the denoised target image. By using the improved target image $y^{k}$, the network is not trained toward the given noisy image $x_{0}$ any longer, which results in a better denoised output image. Figure 1 shows the working flow of the proposed method. Here, the orange bullets and the solid lines refer to the neural network used in the computation with the specific parameters of $\theta_{0},\theta_{1},\theta_{2},\ldots$, and the dashed lines refer to the inputs to the network. Again, it can be observed that $z_{c}$ remains constant, while $z_{v_{P}}$, $z_{v_{P+1}},\ldots$ are changing over the time.

Next, we give mathematical justifications on the joint demosaicing and denoising property of the variational DIP. First, the reason which explains why the DIP performs demosaicing can be found in [21], where it is proven that an auto-encoder with an insufficient number of input channels performs an approximation of the target signal under the Hankel structured low-rank constraint:

(4) $$\begin{array}{cc} \min_{f\in R^{n}} & {∥f^{*}-f∥}^{2}\\ \;\mathrm{subject}\;\mathrm{to}\; & \mathrm{RANK}(H_{d|p}\left(f\right))\leq r<pd \end{array}$$

where $H_{d|p}\left(f\right)$ denotes the Hankel matrix of *f* with *p* input channels and a convolution filter of size *d*, and $f^{*}$ is the target signal. The above approximation can be easily extended to the two-dimensional case. Thus, letting $f=f_{\theta }\left(z_{c}\right)$ and $f^{*}=y^{k}$ for the two-dimensional case, we see that we get a low rank approximation of $y^{k}$. It has been already shown in [22], that a low rank approximation can perform a reconstruction of missing pixels. When applied to the CFA patterned image, this results in a demosaicing. The Hankel structured low-rank approximation in Equation (4) performs a better approximation than the method in [22], since in [22] the low rank approximation is with respect to the Fourier basis, whereas in Equation (4) this is with respect to a learned basis which best reconstructs the given image, and therefore, yields a better demosaicing result.

Now, to consider the effect of adding the varying noise $z_{v}$ to the constant noise $z_{c}$, we consider a multi-variable vector-valued function $f_{\theta }\left(z\right)$ which can be expressed as a set of *M* multi-variable scalar-valued function $f_{\theta_{i}}\left(z\right)$:

$$f_{\theta }\left(z\right)={\left[f_{\theta_{1}}\left(z\right),\cdots ,f_{\theta_{M}}\left(z\right)\right]}^{T}.$$

The Taylor expansion of the *i*-th (i=1,⋯,M) component is

(5) $$f_{\theta_{i}}\left(z+\delta z\right)=f_{\theta_{i}}\left(z\right)+g^{T}\delta z+\frac{1}{2}{\delta z}^{T}H\delta z+\epsilon \left({\delta z}^{3}\right)$$

where $\delta z={\left[{\delta z}_{1},\cdots ,{\delta z}_{N}\right]}^{T}$ is a vector. The gradient vector g and the Hessian matrix H are the first and second order derivatives of the function $f_{\theta_{i}}\left(z\right)$ defined as:

$$g=g_{f_{\theta_{i}}}\left(z\right)=\nabla f_{\theta_{i}}\left(z\right)=\frac{d}{dz}f_{\theta_{i}}\left(z\right)=\left[\begin{array}{c} \frac{\partial f_{\theta_{i}}}{\partial z_{1}}\\ \vdots \\ \frac{\partial f_{\theta_{i}}}{\partial z_{N}} \end{array}\right]$$

$$H=H_{f_{\theta_{i}}}\left(z\right)=\frac{d}{dz}g_{f_{\theta_{i}}}\left(z\right)=\left[\begin{array}{ccc} \frac{\partial^{2}f_{\theta_{i}}}{\partial^{2}z_{1}} & \cdots & \frac{\partial^{2}f_{\theta_{i}}}{\partial z_{1}\partial z_{N}}\\ \vdots & \ddots & \vdots \\ \frac{\partial^{2}f_{\theta_{i}}}{\partial z_{N}\partial z_{1}} & \cdots & \frac{\partial^{2}f_{\theta_{i}}}{\partial^{2}z_{N}} \end{array}\right].$$

We can extend Equation (5) to $f_{\theta }\left(z\right)$ as a vector form. The first two terms can be written as

$$f_{\theta }\left(z+\delta z\right)\approx \left[\begin{array}{c} f_{\theta_{1}}\left(z\right)\\ \vdots \\ f_{\theta_{M}}\left(z\right) \end{array}\right]+\left[\begin{array}{ccc} \frac{\partial f_{\theta_{1}}}{\partial z_{1}} & \cdots & \frac{\partial f_{\theta_{1}}}{\partial z_{N}}\\ \vdots & \ddots & \vdots \\ \frac{\partial f_{\theta_{M}}}{\partial z_{1}} & \cdots & \frac{\partial f_{\theta_{M}}}{\partial z_{N}} \end{array}\right]\left[\begin{array}{c} \delta z_{1}\\ \vdots \\ \delta z_{N} \end{array}\right]=f_{\theta }\left(z\right)+J_{f_{\theta }}\left(z\right)\delta z$$

where $J_{f_{\theta }}\left(z\right)$ is the Jacobian matrix defined over $f_{\theta }\left(z\right)$. The second order term requires a tensor form to be expressed which is difficult to write in vector form, and therefore, we replace the second order term with the error term $\epsilon (∥z_{v}{∥}^{2})$. Then, we can express $f_{\theta }\left(z_{c}+z_{v}\right)$ by the Taylor expansion

$$f_{\theta }\left(z_{c}+z_{v}\right)=f_{\theta }\left(z_{c}\right)+J_{f_{\theta }}\left(z_{c}\right)z_{v}+\epsilon (∥z_{v}{∥}^{2}).$$

This results in

(6) $$\begin{array}{cc} \left|f_{\theta }\left(z_{c}+z_{v_{2}}\right)-f_{\theta }\left(z_{c}+z_{v_{1}}\right)\right| & =|J_{f_{\theta }}\left(z_{c}\right)\left(z_{v_{2}}-z_{v_{1}}\right)+\epsilon (∥z_{v_{2}}{∥}^{2})-\epsilon (∥z_{v_{1}}{∥}^{2})|\\ & =M∥z_{v_{2}}-z_{v_{1}}{∥}_{2} \end{array}$$

where

(7) $$M=\left|J_{f_{\theta }}\left(z_{c}\right)\frac{\left(z_{v_{2}}-z_{v_{1}}\right)}{∥z_{v_{2}}-z_{v_{1}}{∥}_{2}}+\frac{\epsilon (∥z_{v_{2}}{∥}^{2})}{∥z_{v_{2}}-z_{v_{1}}{∥}_{2}}-\frac{\epsilon (∥z_{v_{1}}{∥}^{2})}{∥z_{v_{2}}-z_{v_{1}}{∥}_{2}}\right|$$

and $J_{f_{\theta }}\left(z\right)$ is the Jacobian matrix defined over the vector function $f_{\theta }\left(z\right)$:

$$J_{f_{\theta }}\left(z\right)=\frac{d}{dz}f_{\theta }\left(z\right)$$

Equation (6) implies the fact that if M≠0 and $z_{v_{2}}\neq z_{v_{1}}$, then $f_{\theta }\left(z_{c}+z_{v_{2}}\right)\neq f_{\theta }\left(z_{c}+z_{v_{1}}\right)$; i.e., the outputs $f_{\theta }\left(z_{c}+z_{v_{2}}\right)$ and $f_{\theta }\left(z_{c}+z_{v_{1}}\right)$ cannot be the same. This is contradictory to the loss function in Equation (2), which minimization forces the outputs $f_{\theta }\left(z_{c}+z_{v_{k}}\right)$ to converge to the same image $y^{0}$ for all different inputs $z_{v_{k}},k\geq P$. It should be noted that, with very high probability, M≠0 and $z_{v_{1}}\neq z_{v_{2}}$, since $z_{v_{1}}$ and $z_{v_{2}}$ are random noises. Therefore, the different inputs of $z_{v_{k}}$ act as regularizers which eliminate the components with small $L_{2}$ norm energy from $f_{\theta }\left(z_{c}+z_{v_{k}}\right)$. As the components with small energy will be mostly the noise, this will result in a noise removal of $f_{\theta }\left(z_{c}+z_{v_{k}}\right)$.

Furthermore, if we take the expectation of the different outputs $f_{\theta }\left(z_{c}+z_{v_{k}}\right)$ with respect to $z_{v_{k}}$, we get

(8) $$\begin{array}{cc} E_{z_{v_{k}}}\left\{f_{\theta }\left(z_{c}+z_{v_{k}}\right)\right\} & \approx E_{z_{v_{k}}}\left\{f_{\theta }\left(z_{c}\right)\right\}+E_{z_{v_{k}}}\left\{J_{f_{\theta }}\left(z_{c}\right)z_{v_{k}}\right\}\\ & =E_{z_{v_{k}}}\left\{f_{\theta }\left(z_{c}\right)\right\}+J_{f_{\theta }}\left(z_{c}\right)E_{z_{v_{k}}}\left\{z_{v_{k}}\right\}=E_{z_{v_{k}}}\left\{f_{\theta }\left(z_{c}\right)\right\}=f_{\theta }\left(z_{c}\right), \end{array}$$

which shows the fact that if we put $z_{c}$ as the input after the DIP has been trained, the output $f_{\theta }\left(z_{c}\right)$ will be approximately the average of the outputs $f_{\theta }\left(z_{c}+z_{v_{k}}\right)$ for different $z_{v_{k}}$. This averaging has a further denoising effect which will remove the remaining noise.

The fact that different inputs of $z_{v_{k}}$ result in different outputs can also be shown by the mean value theorem. According to the mean value theorem, there always exists a point $\tilde{z}$ between $z_{v_{1}}$ and $z_{v_{2}}$ such that the following equality holds:

(9) $$f_{\theta }\left(z_{c}+z_{v_{1}}\right)-f_{\theta }\left(z_{c}+z_{v_{2}}\right)=\nabla_{z}f_{\theta }\left(\tilde{z}\right)\cdot \left(z_{v_{1}}-z_{v_{2}}\right).$$

When $z_{v_{1}}\neq z_{v_{2}}$, then the right-hand side of Equation (9) is, with very high probability, non-zero, since it is almost unlikely that $\nabla_{z}f_{\theta }\left(\tilde{z}\right)$ and $\left(z_{v_{1}}-z_{v_{2}}\right)$ are orthogonal to each other. Therefore, with very high probability,

(10) $$|f_{\theta }\left(z_{c}+z_{v_{1}}\right)-f_{\theta }\left(z_{c}+z_{v_{2}}\right)\left|=\right|\nabla_{z}f_{\theta }\left(\tilde{z}\right)\left|\right|z_{v_{1}}-z_{v_{2}}|cosine\left(\gamma \right)\neq 0,$$

where γ is the angle between $\nabla_{z}f_{\theta }\left(\tilde{z}\right)$ and $z_{v_{1}}-z_{v_{2}}$. This means that if there is a difference between the inputs, then the outputs of the DIP cannot be the same, so there will be an averaging which removes the noise.

**Figure 2:**
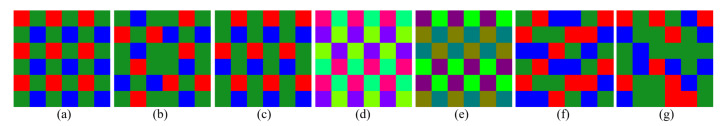
Different color filter arrays (CFAs): (**a**) Bayer CFA [26]; (**b**) Fuji X-Trans CFA [27]; (**c**) Lukac [23]; (**d**) Hirakawa Pattern-A [25]; (**e**) Hirakawa Pattern-B [25]; (**f**) proposed CFA (1:1:1); (**g**) proposed CFA (1:2:1).

Next, we propose a CFA pattern, which we think works well with the proposed demosaicing method. The proposed CFA consists of randomly distributed pixels, where the pixels corresponding to the R, G and B channels take up 33% of the whole CFA pattern each. The design of the proposed CFA is not based on a rigorous analysis, as done in classical CFA designs [23,24,25], but on simple reasoning and experimental results. We reason that if the filters are to learn to generate the R, G and B pixels without any bias for a specific color or a specific position, the best training method would be to train the filters to generate any random color at any random position. For example, if the CFA pattern has, for example, 50% green pixels, as in the Bayer format, the convolutional filters will be trained mainly how to generate the green pixels from the noise. When trained like this, the same convolutional filters may be less effective in generating the R or B pixels. Therefore, we reason that the amount of information should be the same for all three channels; i.e., the CFA should consist of 33% of R, G and B pixels each. In the same manner, we reason that if the filters are to learn to generate the R, G and B pixels without any bias for a specific position, it would be good to train the filters to generate any random color at any random position, which is why we propose a pattern with randomly distributed color pixels. Experimental results show that the randomly patterned CFA works better with the proposed demosaicing method than the Bayer pattern or the Fuji X-Trans pattern. Figure 2 shows the different color filter arrays (CFAs) which are used in the experiments including the proposed CFA.

## 4. Experimental Results

We compared the proposed method with other deep-learning-based demosaicing methods on three different datasets. We added different noises generated from Gaussian distributions with different standard deviations of $\sigma_{R}$, $\sigma_{G}$ and $\sigma_{B}$, for the R, G and B channels, respectively. This is due to the fact that the R, G and B filters absorb different light energy. We compared the proposed method with the method in [28] as a representative of the non-deep learning demosaicing method; the sequential energy minimization (SEM) method [14]; the DemosaicNet [15] with two different CFAs, i.e., the DemosaicNet with Bayer CFA(DNetB) and the DemosaicNet with the Fuji X-Trans CFA(DNetX); and the plain DIP [19]. We made quantitative comparisons with the PSNR (peak signal-to-noise ratio), the CPSNR (color peak signal-to-noise ratio), the SSIM (structural similarity index), the FSIM (feature similarity index) and the FSIMc (feature similarity index chrominance) measures and summarized the results in Tables 2–4. The values in the tables are the average values for the Kodak images and the McMaster (also known as the IMAX) images, respectively. Furthermore, the values corresponding to the red, green and blue channels are the average values for those particular channels, respectively. The parameters for α and β in Equation (3) were set to 0.003 and 0.007, respectively, throughout all the experiments.

Table 1 shows the results of performance comparison when the proposed method was applied for the different CFA patterns; i.e, the Bayer [26], the Fuji X-Trans [27], the Lucak [23], the Hirakawa [25] and the proposed CFA patterns with different noise levels. For the Hirakawa CFA we used the pattern-A and pattern-B patterns which have RGB ratios of 1:1:1 and 1:2:1, respectively. Likewise, for the proposed random patterned CFA, we used the Random1 pattern (RGB ratio of 1:1:1) and the Random2 pattern (RGB ratio of 1:2:1). The CPSNR, SSIM and FSIMc values are the average values of the images in the Kodak image dataset. When the noise is low, the Hirakawa pattern-A CFA shows the largest CPSNR and SSIM values. However, when the noise increases, the proposed random pattern shows larger PSNR, SSIM and FSIM values. This is maybe due to the fact that when the noise increases, the tasks of demosaicing and denoising become similar—i.e., the task of removing the random noise and the task of filling in random colors become similar—so the finding of the parameters which do the demosaicing and denoising tasks simultaneously becomes an easier task with the proposed random CFA than with other CFAs. It can be seen that the proposed CFA pattern mostly shows the largest value, especially when the noise is large.

**Figure 3:**
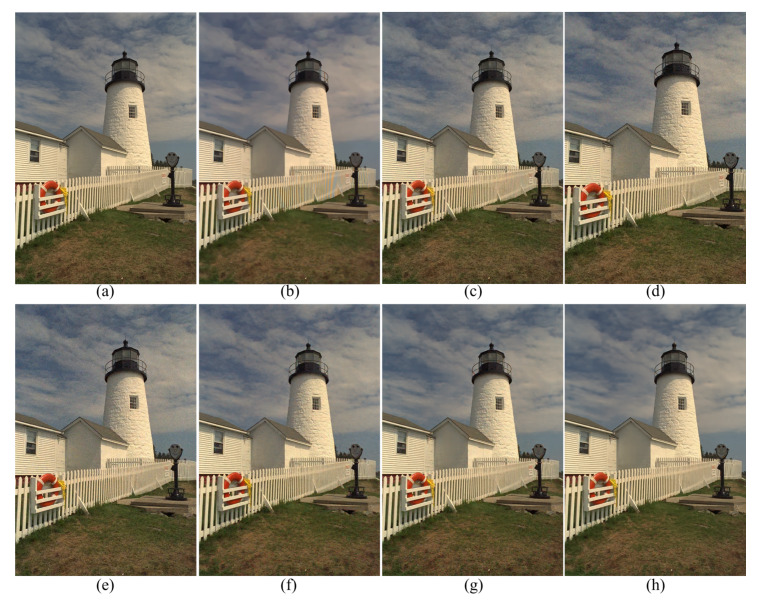
Reconstruction results for the Kodak number 19 image with noise levels $\sigma_{R}=9.83$, $\sigma_{G}=6.24$ and $\sigma_{B}=6.84$. (**a**) Original, (**b**) ADMM, [28] (**c**) SEM, [14] (**d**) DNetB, [15] (**e**) DNetX, [15] (**f**) DIP, [19] (**g**) proposed and (**h**) proposed + BM3D.

**Figure 4:**
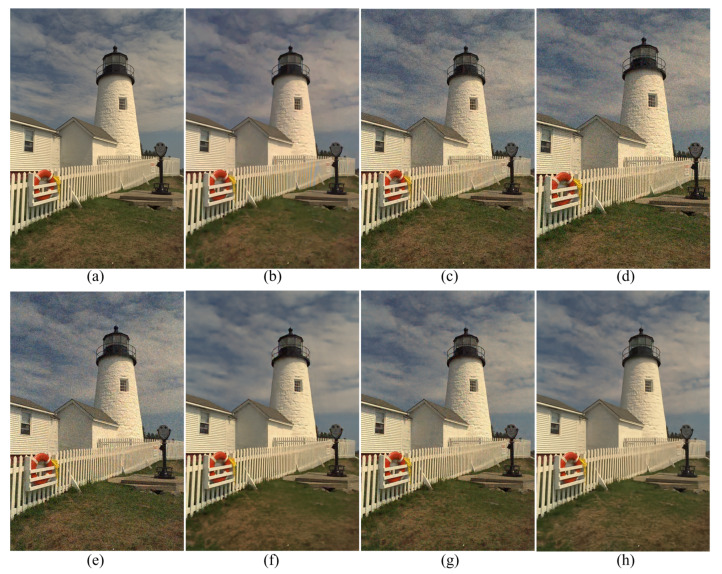
Reconstruction results for the Kodak number 19 image with noise levels $\sigma_{R}=19.67$, $\sigma_{G}=12.48$ and $\sigma_{B}=13.67$. (**a**) Original, (**b**) ADMM, [28] (**c**) SEM, [14] (**d**) DNetB, [15] (**e**) DNetX, [15] (**f**) DIP, [19] (**g**) proposed and (**h**) proposed + BM3D.

**Figure 5:**
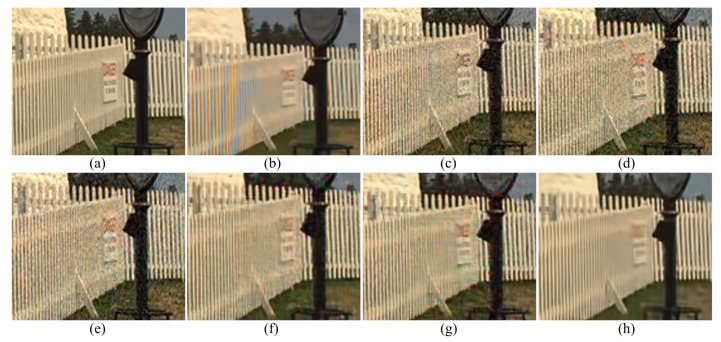
Enlarged regions of Figure 4. (**a**) Original, (**b**) ADMM, [28] (**c**) SEM, [14] (**d**) DNetB, [15] (**e**) DNetX, [15] (**f**) DIP, [19] (**g**) proposed and (**h**) proposed + BM3D.

**Figure 6:**
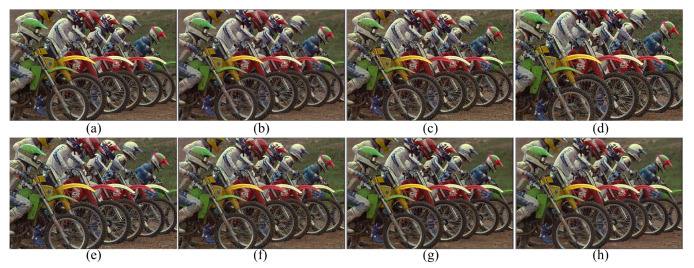
Reconstruction results for the Kodak number 5 image with noise levels $\sigma_{R}=26.22$, $\sigma_{G}=16.64$ and $\sigma_{B}=18.23$. (**a**) Original, (**b**) ADMM, [28] (**c**) SEM, [14] (**d**) DNetB, [15] (**e**) DNetX, [15] (**f**) DIP, [19] (**g**) proposed and (**h**) proposed + BM3D.

**Figure 7:**
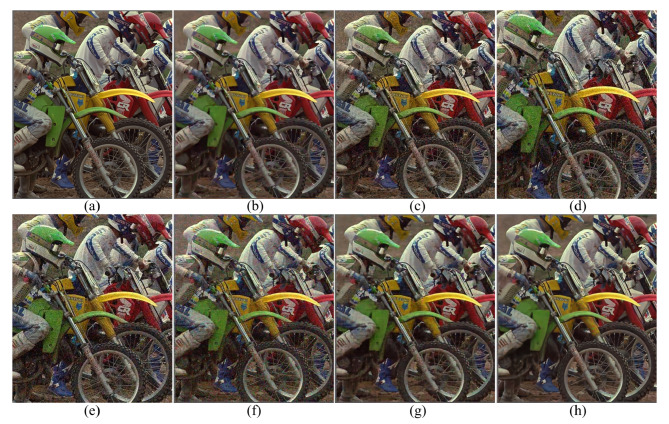
Enlarged regions of Figure 6. (**a**) Original, (**b**) ADMM, [28] (**c**) SEM, [14] (**d**) DNetB, [15] (**e**) DNetX, [15] (**f**) DIP, [19] (**g**) proposed and (**h**) proposed + BM3D.

**Figure 8:**
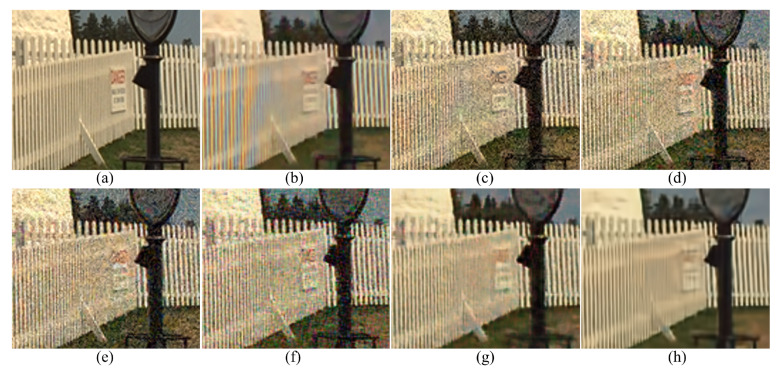
Enlarged regions of the denoising results on the Kodak number 19 image with noise levels $\sigma_{R}=26.22$, $\sigma_{G}=16.64$ and $\sigma_{B}=18.23$. (**a**) Original, (**b**) ADMM, [28] (**c**) SEM, [14] (**d**) DNetB, [15] (**e**) DNetX, [15] (**f**) DIP, [19] (**g**) proposed and (**h**) proposed + BM3D.

Figure 3 shows the results of the different demosaicing methods on the first dataset with color noise of standard deviations $\sigma_{R}=9.83$, $\sigma_{G}=6.24$ and $\sigma_{B}=6.84$ for the R, G and B channels, respectively. The parameter *P* in Equation (3) is set to 1200 for the experiments with this color noise, and to 500 for all the other experiments. When the noise is light, the SEM, the DNetB and the DNetX also produce good joint demosaicing and denoising results. The SEM shows the best quantitative results in the PSNR values for the Kodak dataset, as can be seen in Table 2. However, the proposed method achieves the best results in the SSIM and the FSIM measures for all datasets, and the best PSNR values for the McMaster dataset. Figure 4 and Figure 5 and Table 2, Table 3 and Table 4 show the results on the dataset with color noise of standard deviations $\sigma_{R}=19.67$, $\sigma_{G}=12.48$ and $\sigma_{B}=13.67$. The ADMM method got the highest value of cPSNR for the McMaster dataset; that was due to the fact that the ADMM method incorporates the powerful BM3D denoising [29] and the total variation minimization into a single framework, which results in a large denoising power. Therefore, we experimented also on a combination of the proposed method and the BM3D. In this case, the proposed training method can focus more on finding the parameters for the demosaicing task, leaving a large part of denoising to the BM3D, which results in finding effective parameters for demosaicing. The results of the SEM, DNetB and DNetX are those without using the external BM3D denoising method. The proposed + BM3D outperforms the other methods on the Kodak dataset with respect to the PSNR and SSIM measures. As the noise increases, the ADMM, SEM, DNetB and DNetX result in severe color artifacts, as can be observed from the fence regions in the enlarged images in Figure 5b–e. However, the DIP and the proposed method overcome such color artifacts due to the inherent rank minimization property. The figures are selected according to the best PSNR values, which is why the figures for the DIP are a little more blurry than the figures for the proposed method. The DIP reconstructs the noise when reconstructing the high frequency components while the proposed method does not. Finally, Figure 6 and Figure 7 and Table 2, Table 3 and Table 4 show the results on the dataset with color noise of standard deviations $\sigma_{R}=26.22$, $\sigma_{G}=16.64$ and $\sigma_{B}=18.23$. For this dataset, the non-deep-learning ADMM method outperforms all the deep-learning-based methods, including the proposed method, in the quantitative measures. However, the proposed method outperforms all other deep-learning-based methods. Furthermore, while the ADMM shows large aliasing artifacts, as can be seen in Figure 8b, the proposed method is free from such artifacts. Again, it should be taken into account that this is the result of training with the noisy pattern CFA image only. Furthermore, we fixed all the hyper-parameters of the network for all the different noise levels, which means that the proposed method is not sensitive to the noise levels.

**Figure 9:**
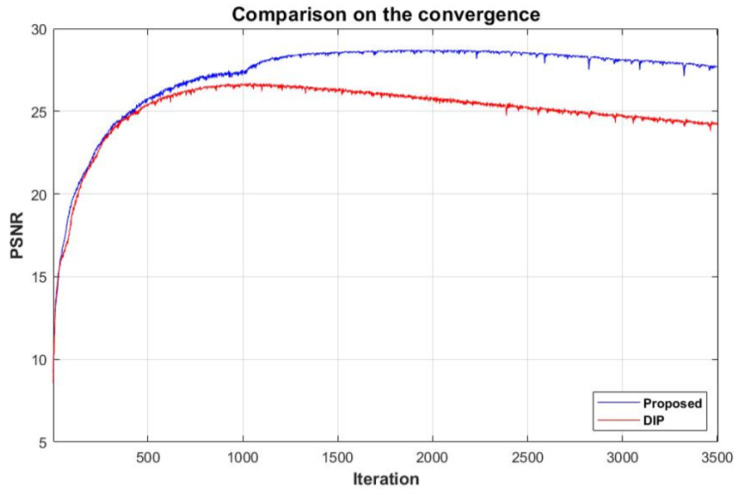
Comparison of the convergence between the DIP and the proposed variational DIP.

Figure 9 compares the convergence of the PSNR values according to the training iterations of the plain DIP and the proposed variational DIP, respectively. As can be seen, the plain DIP converges to a lower PSNR value as the training step iterates, which is due to the fact that the noise in the target image is reconstructed. In comparison, with the proposed variational DIP, the noise is not reconstructed, due to the reasons explained in the previous section. Therefore, the final output image converges to a joint demosaiced and denoised image, which results in a convergence to a higher PSNR value.

Table 5 shows the computational time costs of the different methods. All the methods have been run on a PC with an Intel Core i9-9900K Processor, NVIDIA GeForce RTX 2080 Ti and 32 GB RAM. The proposed method is the slowest of all the methods, which is due to the fact that the proposed method uses a training step for each incoming CFA image. The computational time can be reduced if the proposed method is combined with the meta learning approach. One of the possible methods would be to initialize the neural network with good initial parameters obtained by some pre-training with many images. This should be one of the major topics of further studies.

## 5. Conclusions

In this paper, we proposed a variational deep image prior for the joint demosaicing and denoising of the proposed random color filter array. We mathematically explained why the variational model results in a demosaicing and denoising result, and experimentally verified the performance of the proposed method. The experimental results showed that the proposed method is superior to other deep-learning-based methods, including the deep image prior network. How to apply the proposed method on the demosaicing of color filter arrays including channels other than the three primary color channels could be the topic of further studies. 

## Figures and Tables

**Table 1:** Comparison of the CPSNR, SSIM and FSIMc values for the different CFA patterns used with the proposed method on the Kodak image dataset.

Noise Level	Measure	Bayer	Xtrans	Lukac	HirakawaA	HirakawaB	Random1	Random2
	CPSNR	31.4386	32.1435	31.8650	**32.5018**	32.0552	32.1918	32.1410
$\sigma_{R}$ = 9.83	SSIM-R	0.8543	0.8719	0.8596	**0.8827**	0.8750	0.8718	0.8709
$\sigma_{G}$ = 6.24	SSIM-G	0.8870	0.8769	0.8839	**0.8972**	0.8823	0.8861	0.8863
$\sigma_{B}$ = 6.84	SSIM-B	0.8530	0.8744	0.8590	0.8854	**0.8863**	0.8719	0.8749
	FSIMc	0.9771	**0.9789**	0.9759	0.9368	0.9740	0.9775	0.9775
	CPSNR	29.2582	29.3572	29.4057	**29.7132**	29.5584	29.3914	29.3710
$\sigma_{R}$ = 19.67	SSIM-R	0.7724	0.7719	0.7745	0.8257	**0.8275**	0.7750	0.7927
$\sigma_{G}$ = 12.48	SSIM-G	0.8217	0.8207	0.8244	**0.8452**	0.8402	0.8230	0.8206
$\sigma_{B}$ = 13.67	SSIM-B	0.7948	0.7992	0.7992	**0.8328**	0.8239	0.8028	0.8084
	FSIMc	0.9537	0.9541	**0.9548**	0.9532	0.9516	**0.9548**	**0.9548**
	CPSNR	28.0037	28.0340	28.0733	27.9030	27.5895	27.9799	**28.2210**
$\sigma_{R}$ = 26.22	SSIM-R	0.7184	0.7126	0.7180	**0.7682**	0.7631	0.7127	0.7013
$\sigma_{G}$ = 16.64	SSIM-G	0.7801	0.7743	0.7782	0.7682	0.7785	0.7751	**0.7809**
$\sigma_{B}$ = 18.23	SSIM-B	0.7556	0.7517	0.7543	0.7645	0.7596	0.7542	**0.7670**
	FSIMc	0.9364	0.9354	0.9358	0.9415	**0.9616**	0.9354	0.9372

**Table 2:** Comparison of the PSNR values among the various demosaicing methods on the Kodak and the McMaster image datasets.

Noise Level	Dataset	Measure	ADMM	SEM	DNetB	DNetX	DIP	Proposed	Proposed +BM3D
	**Kodak**	cPSNR	31.3370	32.5468	31.4053	31.3766	30.4512	32.1410	**32.6103**
		PSNR-R	30.9196	31.6351	30.3037	30.4766	29.5487	31.5406	**32.2743**
$\sigma_{R}$ = 9.83		PSNR-G	31.9553	**33.2664**	32.1838	32.0917	31.1663	32.5779	32.8726
$\sigma_{G}$ = 6.24		PSNR-B	31.2722	**32.9731**	32.0131	31.7628	30.9852	32.4079	32.7272
$\sigma_{B}$ = 6.84	**McMaster**	cPSNR	32.1258	30.7908	31.1171	31.0155	29.6490	32.6603	**33.0659**
		PSNR-R	31.8458	29.7472	29.9868	29.9864	28.7221	32.2605	**32.9728**
		PSNR-G	33.2163	32.1750	32.2558	32.1366	30.1651	33.1813	**33.4180**
		PSNR-B	31.6661	30.9114	31.5195	31.2861	30.4179	32.8239	**33.0647**
	**Kodak**	cPSNR	30.1883	26.1682	25.9266	25.8636	27.2001	29.3710	**30.2026**
		PSNR-R	29.5430	25.1485	24.5928	24.9497	26.1787	28.7261	**29.6902**
$\sigma_{R}$ = 19.67		PSNR-G	**30.7728**	26.4776	26.6385	26.2463	27.8791	29.9587	30.4681
$\sigma_{G}$ = 12.48		PSNR-B	30.3904	27.1772	26.9603	26.5889	27.9003	29.9483	**30.5204**
$\sigma_{B}$ = 13.67	**McMaster**	cPSNR	**30.5794**	25.7280	26.1234	26.0321	26.7356	29.7984	30.4533
		PSNR-R	29.9344	24.4154	24.6482	24.8721	25.5419	28.9401	**29.9461**
		PSNR-G	**31.5879**	26.5727	27.0705	26.6617	27.3464	30.4840	30.9327
		PSNR-B	30.5402	26.6814	27.2079	26.9079	27.8159	30.3375	**30.7383**
	**Kodak**	cPSNR	**29.3247**	23.2843	23.4976	23.4724	25.6570	28.2210	29.0672
		PSNR-R	**28.5560**	22.3131	22.0491	22.5529	24.7341	27.3588	28.4363
$\sigma_{R}$ = 26.22		PSNR-G	**29.9424**	23.5352	24.2448	23.7847	26.1365	28.7616	29.3628
$\sigma_{G}$ = 16.64		PSNR-B	**29.6403**	24.3010	24.6938	24.2836	26.2839	28.8031	29.5067
$\sigma_{B}$ = 18.23	**McMaster**	cPSNR	**29.5312**	23.2554	23.8799	23.7807	25.4785	28.2050	29.0961
		PSNR-R	**28.6716**	21.8944	22.2896	22.6013	24.2930	27.1305	28.4053
		PSNR-G	**30.5938**	23.9948	24.8180	24.2958	26.0823	29.0621	29.6725
		PSNR-B	**29.6901**	24.3931	25.1801	24.8034	26.5224	28.9340	29.4845

**Table 3:** Comparison of the SSIM values among the various demosaicing methods on the Kodak and the McMaster image datasets.

Noise Level	Dataset	Measure	ADMM	SEM	DNetB	DNetX	DIP	Proposed	Proposed +BM3D
	**Kodak**	SSIM-R	0.8613	0.8581	0.7774	0.7834	0.7977	0.8709	**0.8871**
$\sigma_{R}$ = 9.83		SSIM-G	0.8840	0.8785	0.8267	0.8154	0.8625	0.8863	**0.8891**
$\sigma_{G}$ = 6.24		SSIM-B	0.8524	**0.8802**	0.8288	0.8209	0.8469	0.8749	0.8794
$\sigma_{B}$ = 6.84	**McMaster**	SSIM-R	0.8846	0.8016	0.7612	0.7662	0.8017	0.8805	**0.8993**
		SSIM-G	**0.9132**	0.8577	0.8222	0.8134	0.8657	0.9013	0.9074
		SSIM-B	0.8624	0.8192	0.8068	0.8000	0.8407	0.8758	**0.8822**
	**Kodak**	SSIM-R	0.8253	0.5630	0.5372	0.5405	0.6490	0.7927	**0.8300**
$\sigma_{R}$ = 19.67		SSIM-G	**0.8535**	0.6007	0.6087	0.5762	0.7501	0.8206	0.8372
$\sigma_{G}$ = 12.48		SSIM-B	0.8264	0.6256	0.6218	0.5931	0.7320	0.8084	**0.8270**
$\sigma_{B}$ = 13.67	**McMaster**	SSIM-R	**0.8416**	0.5258	0.5320	0.5385	0.6529	0.8104	0.8378
		SSIM-G	**0.8817**	0.6083	0.6185	0.5919	0.7725	0.8467	0.8588
		SSIM-B	**0.8288**	0.5941	0.6209	0.6007	0.7485	0.8241	0.8221
	**Kodak**	SSIM-R	**0.7972**	0.4296	0.4240	0.4319	0.5881	0.7013	0.7948
$\sigma_{R}$ = 26.22		SSIM-G	**0.8315**	0.4642	0.4972	0.4657	0.6875	0.7809	0.8074
$\sigma_{G}$ = 16.64		SSIM-B	**0.8058**	0.4858	0.5093	0.4819	0.6706	0.7670	0.7978
$\sigma_{B}$ = 18.23	**McMaster**	SSIM-R	**0.8091**	0.4066	0.4289	0.4364	0.5915	0.7463	0.7961
		SSIM-G	**0.8579**	0.4828	0.5173	0.4864	0.7156	0.8116	0.8265
		SSIM-B	**0.8031**	0.4672	0.5213	0.4962	0.6863	0.7839	0.7873

**Table 4:** Comparison of the FSIM values among the various demosaicing methods on the Kodak and the McMaster image datasets.

Noise Level	Dataset	Measure	ADMM	SEM	DNetB	DNetX	DIP	Proposed	Proposed +BM3D
	**Kodak**	FSIMc	0.9722	**0.9792**	0.9743	0.9730	0.9666	0.9775	0.9764
		FSIM-R	0.9580	0.9635	0.9469	0.9471	0.9469	0.9665	**0.9705**
$\sigma_{R}$ = 9.83		FSIM-G	0.9729	**0.9794**	0.9738	0.9734	0.9639	0.9736	0.9737
$\sigma_{G}$ = 6.24		FSIM-B	0.9574	**0.9728**	0.9650	0.9607	0.9606	0.9704	0.9708
$\sigma_{B}$ = 6.84	**McMaster**	FSIMc	0.9807	0.9788	0.9756	0.9746	0.9708	0.9824	**0.9824**
		FSIM-R	0.9650	0.9565	0.9478	0.9481	0.9540	0.9716	**0.9756**
		FSIM-G	**0.9802**	0.9773	0.9743	0.9743	0.9667	0.9779	0.9784
		FSIM-B	0.9629	0.9600	0.9613	0.9587	0.9638	0.9749	**0.9754**
	**Kodak**	FSIMc	**0.9598**	0.9232	0.9225	0.9175	0.9276	0.9548	0.9562
		FSIM-R	0.9399	0.8936	0.8654	0.8738	0.8978	**0.9548**	0.9463
$\sigma_{R}$ = 19.67		FSIM-G	**0.9603**	0.9266	0.9217	0.9210	0.9270	0.9514	0.9540
$\sigma_{G}$ = 12.48		FSIM-B	0.9471	0.9259	0.9068	0.9014	0.9245	0.9490	**0.9520**
$\sigma_{B}$ = 13.67	**McMaster**	FSIMc	**0.9695**	0.9290	0.9304	0.9268	0.9378	0.9625	0.9656
		FSIM-R	0.9462	0.8911	0.8749	0.8803	0.9086	0.9467	**0.9525**
		FSIM-G	**0.9683**	0.9315	0.9277	0.9284	0.9345	0.9582	0.9618
		FSIM-B	0.9509	0.9197	0.9085	0.9056	0.9320	0.9554	**0.9591**
	**Kodak**	FSIMc	**0.9502**	0.8811	0.8835	0.8776	0.9007	0.9372	0.9433
		FSIM-R	0.9255	0.8479	0.8148	0.8272	0.8687	0.9042	**0.9305**
$\sigma_{R}$ = 26.22		FSIM-G	**0.9508**	0.8868	0.8848	0.8832	0.8981	0.9352	0.9417
$\sigma_{G}$ = 16.64		FSIM-B	0.9383	0.8876	0.8676	0.8607	0.8969	0.9329	**0.9406**
$\sigma_{B}$ = 18.23	**McMaster**	FSIMc	**0.9601**	0.8919	0.8971	0.8929	0.9164	0.9470	0.9530
		FSIM-R	0.9309	0.8462	0.8291	0.8379	0.8855	0.9258	**0.9351**
		FSIM-G	**0.9588**	0.8982	0.8946	0.8964	0.9132	0.9437	0.9500
		FSIM-B	0.9402	0.8869	0.8727	0.8700	0.9092	0.9403	**0.9473**

**Table 5:** Showing the computational time costs of the different joint demosaicing and denoising methods.

Method	ADMM	SEM	DNetB	DNetX	DIP	Proposed
**Time Cost**	567 s	465 s	8 s	8 s	525 s	647 s
**Software Tool**	MATLAB	Python	Python	Python	Pytorch	Pytorch

## References

[1] Pekkucuksen I., Altunbasak Y. (2013). Multiscale gradients-based color filter array interpolation. IEEE Trans. Image Process..

[2] Kiku D., Monno Y., Tanaka M., Okutomi M. (2016). Beyond color difference: Residual interpolation for color image demosaicking. IEEE Trans. Image Process..

[3] Gunturk B.K., Altunbasak Y., Mersereau R.M. (2002). Color plane interpolation using alternating projections. IEEE Trans. Image Process..

[4] Alleysson D., Süsstrunk S., Hérault J. (2005). Linear demosaicing inspired by the human visual system. IEEE Trans. Image Process..

[5] Gunturk B.K., Glotzbach J., Altunbasak Y., Schafer R.W., Mersereau R.M. (2005). Demosaicking: Color filter array interpolation. IEEE Signal Process. Mag..

[6] Kimmel R. (1999). Demosaicing: Image reconstruction from color ccd samples. IEEE Trans. Image Process..

[7] Pei S.-C., Tam I.-K. (2003). Effective color interpolation in ccd color filter arrays using signal correlation. IEEE Trans. Circuits Syst. Video Technol..

[8] Menon D., Calvagno G. (2011). Color image demosaicking: An overview. Signal Process Image.

[9] Dubois E. (2005). Frequency-domain methods for demosaicking of bayer-sampled color images. IEEE Signal Process. Lett..

[10] Hirakawa K., Parks T.W. (2006). Joint demosaicing and denoising. IEEE Trans. Image Process..

[11] Jeon G., Dubois E. (2013). Demosaicking of noisy bayer sampled color images with least-squares luma-chroma demultiplexing and noise level estimation. IEEE Trans. Image Process..

[12] Buades A., Duran J. (2019). CFA Video Denoising and Demosaicking Chain via Spatio-Temporal Patch-Based Filtering. IEEE Trans. Circuits Syst. Video Technol..

[13] Khashabi D., Nowozin S., Jancsary J., Fitzgibbon A.W. (2014). Joint demosaicing and denoising via learned nonparametric random fields. IEEE Trans. Image Process..

[14] Klatzer T., Hammernik K., Knobelreiter P., Pock T. Learning joint demosaicing and denoising based on sequential energy minimization. Proceedings of the 2016 IEEE International Conference on Computational Photography (ICCP).

[15] Gharbi M., Chaurasia G., Paris S., Durand F. (2016). Deep Joint Demosaicking and Denoising. ACM Trans. Graph. (TOG).

[16] Huang T., Wu F., Dong W., Guangming S., Li X. Lightweight Deep Residue Learning for Joint Color Image Demosaicking and Denoising. Proceedings of the 2018 International Conference on Pattern Recognition (ICPR).

[17] Ehret T., Davy A., Arias P., Facciolo G. Joint Demosaicking and Denoising by Fine-Tuning of Bursts of Raw Images. Proceedings of the 2019 International Conference on Computer Vision.

[18] Kokkinos F., Lefkimmiatis S. (2019). Iterative Joint Image Demosaicking and Denoising Using a Residual Denoising Network. IEEE Trans. Image Process..

[19] Ulyanov D., Vedaldi A., Lempitsky V. Deep Image Prior. Proceedings of the IEEE Conference on Computer Vision and Pattern Recognition (CVPR).

[20] Kingma D.P., Welling M. Auto-Encoding Variational Bayes. Proceedings of the 2nd International Conference on Learning Representations(ICLR 2014).

[21] Ye J.C., Han Y.S. (2017). Deep Convolutional Framelets: A General Deep Learning for Inverse Problems. SIAM J. Imaging Sci..

[22] Zhou J., Kwan C., Ayhan B. A High Performance Missing Pixel Reconstruction Algorithm for Hyperspectral Images. Proceedings of the 2nd International Conference on Applied and Theoretical Information Systems.

[23] Lukac R., Konstantinos N.P. (2005). Color Filter Arrays: Design and Performance Analysis. IEEE Trans. Consum. Electron..

[24] Vaughn I.J., Alenin A.S., Tyo J.S. (2017). Focal plane filter array engineering I: Rectangular lattices. Opt. Express.

[25] Hirakawa K., Wolfe P.J. (2008). Spatio-Spectral Color Filter Array Design for Optimal Image Recovery. IEEE Trans. Image Process..

[26] Bayer B. (1976). Color Imaging Array. U.S. Patent.

[27] Fujifilm X-Pro1. http://www.fujifilmusa.com/products/digital_cameras/x/fujifilm_x_pro1/features.

[28] Tan H., Zeng X., Lai S., Liu Y., Zhang M. Joint demosaicing and denoising of noisy bayer images with ADMM. Proceedings of the 2017 IEEE International Conference on Image Processing (ICIP 2017).

[29] Kostadin D., Alessandro F., Vladimir K., Karen E. (2007). Image denoising by sparse 3D transform-domain collaborative filtering. IEEE Trans. Image Process..

